# Obstructive Sleep Apnea Risk and Incidental Coronary Artery Calcification on Routine Chest Computed Tomography

**DOI:** 10.3390/jcm15062230

**Published:** 2026-03-15

**Authors:** Zeynep Atceken, Sezer Kula, Irem Sena Konakci, Cetin Atasoy, Aylin Pihtili, Yüksel Peker

**Affiliations:** 1 Department of Radiology, Koc University School of Medicine, 34010 Istanbul, Türkiye; zatceken@kuh.ku.edu.tr (Z.A.); ikonakci@kuh.ku.edu.tr (I.S.K.); catasoy@kuh.ku.edu.tr (C.A.); 2 Department of Pulmonary Medicine, School of Medicine, Istanbul University, 34093 Istanbul, Türkiye; draylin02@yahoo.com; 3 Department of Pulmonology, Koc University School of Medicine, 34010 Istanbul, Türkiye; 4 Department of Molecular and Clinical Medicine, Institute of Medicine, Sahlgrenska Academy, University of Gothenburg, 40530 Gothenburg, Sweden; 5 Department of Clinical Sciences, Respiratory Medicine and Allergology, Faculty of Medicine, Lund University, 22100 Lund, Sweden; 6 Division of Pulmonary, Allergy, and Critical Care Medicine, University of Pittsburgh School of Medicine, Pittsburgh, PA 15260, USA

**Keywords:** obstructive sleep apnea, coronary artery calcification, chest computed tomography, subclinical atherosclerosis, opportunistic imaging

## Abstract

**Background:** Obstructive sleep apnea (OSA) is associated with increased cardiovascular morbidity; however, its relationship with subclinical coronary atherosclerosis detected incidentally on routine chest computed tomography (CT) remains incompletely defined. We aimed to evaluate the association between questionnaire-based OSA risk and moderate-to-severe coronary artery calcification (CAC) in patients without known cardiac disease undergoing non-contrast chest CT for non-cardiac indications. **Methods:** In this prospective cross-sectional study, 268 consecutive adults undergoing routine non-contrast chest CT were included. OSA risk was assessed using the Berlin Questionnaire (BQ) and a modified BQ (mBQ), excluding hypertension and obesity components. CAC was quantified using the Agatston method on non-gated CT images, and moderate-to-severe CAC was defined as a score > 100. Multivariable logistic regression models were adjusted for age, sex, smoking status, alcohol use, obesity, lung disease, diabetes mellitus and hypertension. **Results:** Moderate-to-severe CAC was substantially more prevalent among patients at high risk for OSA than among those at low risk (43.1% vs. 12.0%, *p* < 0.001). In unadjusted analyses, high-risk OSA was strongly associated with CAC > 100. After multivariable adjustment, BQ-defined high-risk OSA remained independently associated with moderate-to-severe CAC (adjusted odds ratio [OR] 2.74, 95% confidence interval [CI] 1.29–5.78, *p* = 0.008). Similar results were observed with the mBQ (adjusted OR 2.62, 95% CI 1.27–5.41, *p* = 0.009). Increased snoring intensity was also independently associated with CAC > 100 (adjusted OR 2.25, 95% CI 1.07–4.72, *p* = 0.032). **Conclusions:** Questionnaire-defined high-risk OSA is independently associated with moderate-to-severe incidental CAC detected on routine chest CT. These findings support the integration of sleep-related risk assessment into opportunistic cardiovascular imaging frameworks and highlight the potential role of thoracic CT in multidimensional cardiovascular risk stratification.

## 1. Introduction

Obstructive sleep apnea (OSA) is a highly prevalent and underdiagnosed sleep-related breathing disorder characterized by recurrent episodes of partial or complete upper airway collapse during sleep, resulting in intermittent hypoxia, intrathoracic pressure swings, sleep fragmentation, and sympathetic overactivation. Population-based studies estimate that moderate-to-severe OSA affects a substantial proportion of middle-aged and older adults, with prevalence rising in parallel with increasing obesity rates worldwide [1,2]. Beyond its impact on daytime sleepiness and quality of life, OSA has emerged as a major cardiometabolic disorder with systemic consequences.

The cardiovascular implications of OSA are extensive. Experimental and clinical data have demonstrated that intermittent hypoxia and arousal-induced sympathetic surges trigger oxidative stress, systemic inflammation, endothelial dysfunction, platelet activation, and metabolic dysregulation [3,4,5,6,7,8,9]. These pathophysiological processes promote vascular remodeling and accelerate atherogenesis. OSA has been associated with hypertension, atrial fibrillation, coronary artery disease, heart failure, stroke, and increased cardiovascular mortality [3,6,10,11,12]. Importantly, the relationship between OSA and cardiovascular disease appears to be dose-dependent in several cohorts, suggesting that cumulative nocturnal hypoxic burden may contribute directly to vascular injury [3,11].

Coronary artery calcification (CAC) represents a cumulative marker of coronary atherosclerotic burden and is a robust, independent predictor of future cardiovascular events across diverse populations [13]. The Agatston score, derived from non-contrast cardiac CT, provides a quantitative assessment of calcified plaque burden and improves risk discrimination beyond traditional risk factors. Moderate-to-severe CAC (Agatston score > 100) is widely recognized as a clinically meaningful threshold associated with increased risk of myocardial infarction and cardiovascular death [14]. As such, CAC scoring has become an established tool in preventive cardiology.

Although CAC assessment is traditionally performed using electrocardiogram (ECG)-gated cardiac CT, a growing body of evidence demonstrates that coronary calcification identified incidentally on routine non-gated thoracic CT carries important prognostic information [15,16,17,18,19]. Non-cardiac chest CT examinations are performed in large numbers for pulmonary, oncologic, infectious, and other indications. These examinations frequently capture the coronary arteries within the imaging field of view, thereby providing an opportunity for opportunistic cardiovascular risk stratification. Professional society guidelines and consensus statements increasingly recommend systematic reporting of incidental CAC detected on non-gated chest CT due to its strong association with adverse cardiovascular outcomes [15,18].

The concept of opportunistic imaging leverages routinely acquired diagnostic studies to extract additional clinically relevant information without additional radiation exposure or cost. In this context, identifying subclinical atherosclerosis on chest CT may uncover individuals at elevated cardiovascular risk who would otherwise remain unrecognized. However, imaging findings alone do not provide insight into underlying systemic drivers of atherosclerosis. Integrating imaging biomarkers such as CAC with clinical risk profiles—including sleep-related disorders—may enhance risk stratification and guide preventive interventions.

Multiple imaging studies have explored the association between OSA and coronary atherosclerosis. Prior investigations using ECG-gated CT, coronary CT angiography, and intravascular imaging have demonstrated associations between polysomnography-confirmed OSA and elevated CAC scores, increased non-calcified plaque burden, and high-risk plaque features [20,21,22,23,24,25,26,27,28,29,30,31,32]. More recently, Macek et al. [33] reported that OSA was associated with a higher likelihood of clinically significant CAC assessed non-invasively using calcium scoring techniques. These findings suggest that OSA may contribute to early vascular injury and subclinical atherosclerosis even before the onset of overt cardiovascular disease. However, most of these studies were conducted in selected cardiac imaging cohorts or relied on formal sleep laboratory diagnosis. Such settings differ substantially from routine radiology practice, where OSA is frequently undiagnosed and polysomnography is not readily available.

In parallel, the concept of opportunistic imaging has gained increasing attention in radiology. Opportunistic imaging refers to the extraction of clinically meaningful information from imaging studies performed for unrelated clinical indications. Because millions of chest CT examinations are performed annually worldwide, incidental detection of coronary artery calcification represents a valuable opportunity to identify individuals at increased cardiovascular risk who might otherwise remain undiagnosed. Integrating imaging biomarkers such as CAC with clinical risk indicators—including OSA—may therefore enhance cardiovascular risk stratification in routine clinical practice.

In everyday clinical practice, questionnaire-based screening tools are commonly used to identify individuals at high risk for OSA. The Berlin Questionnaire (BQ) is one of the most widely validated instruments and incorporates snoring characteristics, daytime sleepiness, and the presence of hypertension or obesity [34]. While the BQ performs reasonably well in identifying individuals at risk for moderate-to-severe OSA, its inclusion of hypertension and obesity—both established cardiovascular risk factors—raises important methodological considerations. Specifically, associations between BQ-defined OSA risk and CAC could theoretically reflect the embedded cardiometabolic components rather than OSA-related mechanisms per se.

To address this potential confounding, a modified Berlin Questionnaire (mBQ) excluding hypertension and obesity components has been developed and validated [35]. The mBQ focuses on symptom-based domains such as snoring intensity, witnessed apneas, and excessive daytime or morning sleepiness. Evaluating both the standard and modified definitions allows assessment of whether the association between OSA risk and CAC persists independent of overlapping cardiometabolic risk factors.

Despite accumulating evidence linking OSA to coronary atherosclerosis, data remain limited regarding the relationship between questionnaire-based OSA risk and incidental coronary artery calcification detected on routine non-gated chest CT performed for non-cardiac indications. In routine radiology practice, many individuals undergoing chest CT have undiagnosed OSA, and systematic sleep evaluation is rarely integrated into imaging workflows. Understanding whether simple symptom-based screening tools can identify individuals with increased CAC burden may therefore have important implications for opportunistic cardiovascular risk assessment.

Accordingly, the present prospective cross-sectional study aimed to investigate whether high-risk OSA, assessed using both the standard BQ and the mBQ, is independently associated with moderate-to-severe CAC (Agatston score > 100) detected incidentally on non-contrast chest CT in patients without known cardiac disease [36]. We further examined the independent contribution of snoring intensity as a symptom-level marker of OSA-related burden. By integrating questionnaire-based OSA risk assessment with opportunistic imaging findings, this study seeks to clarify the potential role of thoracic CT as a platform for combined sleep and cardiovascular risk stratification.

## 2. Materials and Methods

### 2.1. Study Design and Population

**Figure 1:**
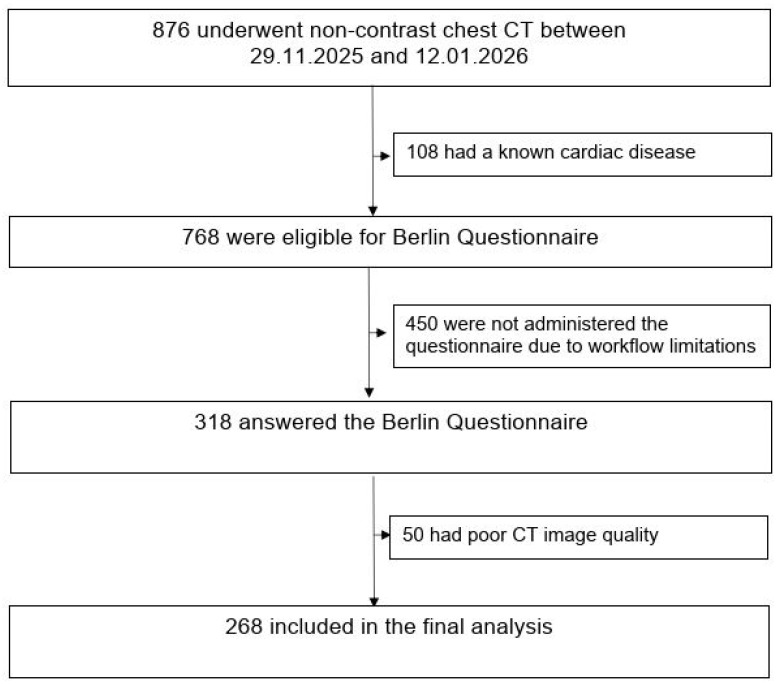
Flow chart illustrating patient selection and final study cohort.

This prospective cross-sectional study included 268 consecutive adult patients who underwent non-contrast chest CT for non-cardiac clinical indications at a single tertiary care center between 29 November 2025 and 12 January 2026. Patients with documented ischemic heart disease were excluded to focus on subclinical coronary atherosclerosis. Documented ischemic heart disease was defined as a prior clinical diagnosis of coronary artery disease, including history of myocardial infarction, coronary revascularization, or physician-documented angina. Patients with previously documented coronary artery disease were excluded based on medical history and electronic medical records. Formal cardiology evaluation was not performed as part of the study protocol. Patients with previously diagnosed OSA receiving active treatment such as continuous positive airway pressure (CPAP) therapy were not included in the study cohort. Demographic and clinical data were obtained from electronic medical records at the time of imaging. Questionnaire administration depended on staff availability during routine clinical workflow; therefore, consecutive eligible patients were approached when feasible. The study population selection process, including inclusion and exclusion criteria and the final analytical sample, is summarized in the flowchart (Figure 1).

Because CT examinations were performed for routine non-cardiac clinical indications, imaging acquisition and interpretation were not influenced by study participation. Questionnaire data were collected on the same day as CT acquisition to ensure temporal consistency between clinical assessment and imaging findings. Patients were approached consecutively during the predefined study period, and no additional clinical interventions were performed as part of the study protocol.

### 2.2. CT Acquisition and Coronary Artery Calcification Assessment

All chest CT examinations were performed using routine non-contrast protocols, acquired in the supine position during a single breath-hold. Chest CT images were reconstructed using a slice thickness of 1 mm with a standard soft-tissue reconstruction kernel. CAC was assessed on non-gated images using dedicated calcium-scoring software (syngo.via VB60 Coronary Calcium Scoring, Siemens Healthineers, Forchheim, Germany). The software identified candidate calcifications using the conventional ≥130 HU threshold and displayed the coronary tree by vessel territory (including the left main coronary artery, the left anterior descending artery, the left circumflex artery, the right coronary artery, and their respective branches) to support visual confirmation and correct vessel assignment.

**Figure 2:**
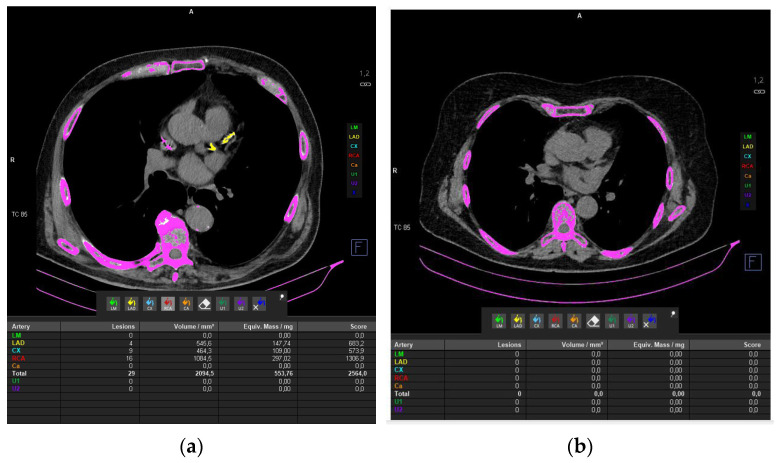
(**a**) shows CT images of the patient with the highest CAC score, whereas (**b**) shows CT images of a patient with a normal calcium score.

High-attenuation foci outside the coronary arteries were excluded, and adjacent non-coronary calcifications inadvertently captured by the automated algorithm (e.g., mitral annular or pericardial calcifications) were manually removed before finalizing the score. Calcium burden was quantified using the Agatston method by summing vessel-level scores to derive the total Agatston score, which was used for statistical analyses [36]. CAC severity was categorized according to the total Agatston score, and moderate-to-severe CAC was defined as >100 in line with established prognostic thresholds, irrespective of the clinical indication for CT. Figure 2a shows CT images of the patient with the highest CAC score, whereas Figure 2b shows CT images of a patient with a normal calcium score.

All calcium scoring analyses were performed by an experienced radiologist with expertise in thoracic imaging who was blinded to OSA risk classification at the time of evaluation. In cases of borderline attenuation values or potential motion-related artifacts, visual confirmation was performed to ensure accurate vessel attribution. For descriptive purposes, CAC scores were additionally stratified into clinically recognized categories (0, 1–100, 101–400, and >400), although the predefined analytical threshold for moderate-to-severe CAC was >100.

### 2.3. OSA Risk Assessment

Risk of OSA was assessed using the BQ, a validated screening tool comprising three symptom-based categories: snoring-related symptoms, daytime sleepiness, and the presence of hypertension or obesity [34]. Patients were classified as high risk for OSA if at least two of the three categories were positive, in accordance with standard scoring criteria.

To minimize the potential confounding related to the inclusion of cardiometabolic factors within the BQ, OSA risk was additionally evaluated using a modified BQ (mBQ), in which the hypertension and obesity components were excluded [35]. The mBQ evaluates three symptom domains: snoring intensity/frequency, witnessed apneas during sleep, and excessive daytime or morning sleepiness. Participants were classified as having high-risk OSA if two or more symptom domains were positive; otherwise, they were classified as low risk.

In symptom-focused analyses, snoring intensity, derived from the snoring-related component of the mBQ, was evaluated separately as an indicator of OSA-related symptom burden.

All questionnaires were administered in person by trained clinical personnel. Participants were instructed to respond based on their habitual sleep patterns over the preceding months rather than isolated recent events. In cases of uncertainty, clarifications were provided without influencing responses. This approach was used to minimize reporting bias and to enhance the internal consistency of symptom-based classification.

### 2.4. Clinical Variables

Collected clinical variables included age, sex, current smoking status, alcohol use, diabetes mellitus, chronic lung disease, hypertension, body mass index (BMI), and obesity status. Hypertension and obesity were defined according to documented clinical diagnoses and standard BMI thresholds, respectively.

### 2.5. Statistical Analysis

Continuous variables are presented as mean ± standard deviation or median with interquartile range, as appropriate, and categorical variables as counts and percentages. Comparisons between groups were performed using Student’s *t* test or Mann–Whitney U test for continuous variables and the χ^2^ test or Fisher’s exact test for categorical variables.

Multivariable logistic regression analyses were used to evaluate the association between the OSA risk and moderate-to-severe CAC. Separate models were constructed for the BQ-defined high-risk OSA and the mBQ-defined high-risk OSA. All models were adjusted for age, sex, smoking status, alcohol use, diabetes mellitus, lung disease, hypertension, and obesity. Snoring intensity was analyzed in a separate multivariable model to avoid construct overlap with composite OSA risk variables. No formal a priori sample size calculation was performed, as this was a prospective observational study including consecutively enrolled patients during a predefined study period. Results are reported as odds ratios (ORs) with 95% confidence intervals (CIs). A two-sided *p* value < 0.05 was considered statistically significant. Statistical analyses were performed using IBM SPSS Statistics for Windows, Version 28.0 (IBM Corp., Armonk, NY, USA).

## 3. Results

### 3.1. Study Population

As shown in Figure 1, the final analytical cohort consisted of 268 patients. The mean age of the overall population was 59.6 ± 13.5 years, and 41.4% were men. The mean BMI was 26.8 ± 5.3 kg/m^2^. Moderate-to-severe CAC was present in 23.9% (64/268) of patients. According to the BQ, 102 patients (38.1%) were classified as high risk for OSA, whereas 166 patients (61.9%) were classified as low risk.

### 3.2. Baseline Characteristics According to the Berlin Questionnaire–Defined OSA Risk

Baseline demographic, clinical, and imaging characteristics stratified by BQ-defined OSA risk are summarized in Table 1. Patients classified as high risk for OSA were significantly older (65.6 ± 11.1 vs. 55.9 ± 13.6 years, *p* < 0.001) and had a higher BMI (28.1 ± 5.4 vs. 25.9 ± 5.0 kg/m^2^, *p* = 0.001) than low-risk patients. The proportion of men was also higher in the high-risk OSA group (52.0% vs. 34.9%, *p* = 0.009).

High-risk OSA patients had a higher prevalence of diabetes mellitus (28.4% vs. 13.3%, *p* = 0.004), hypertension (58.8% vs. 22.9%, *p* < 0.001), obesity (34.3% vs. 14.5%, *p* < 0.001), and chronic lung disease (14.7% vs. 2.4%, *p* < 0.001). There were no significant differences between groups with respect to current smoking status or alcohol use. Moderate-to-severe CAC was substantially more prevalent among patients at high risk for OSA than among those at low risk (43.1% vs. 12.0%, *p* < 0.001).

### 3.3. Association Between OSA Risk and Coronary Artery Calcification

In univariate analyses, patients classified as high risk for OSA according to the BQ demonstrated a markedly higher prevalence of moderate-to-severe CAC compared with low-risk patients (43.1% vs. 12.0%, *p* < 0.001). In unadjusted logistic regression analysis, BQ-defined high-risk OSA was strongly associated with CAC > 100 (OR 5.53, 95% CI 3.00–10.20, *p* < 0.001).

**Figure 3:**
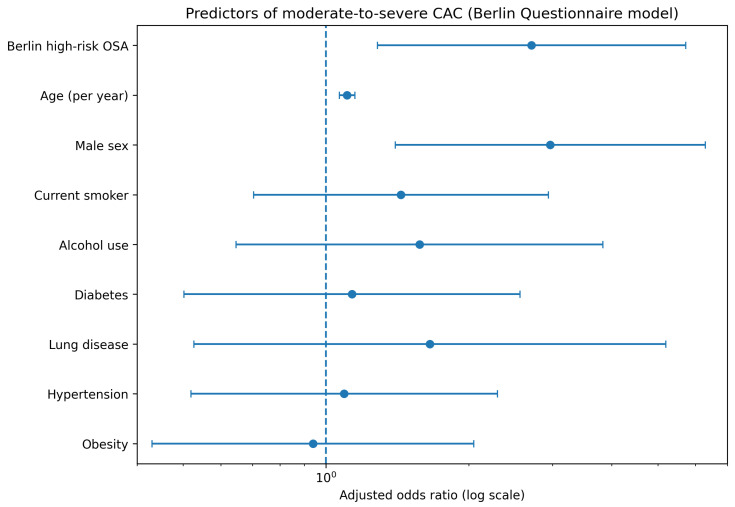
Predictors of moderate-to-severe coronary artery calcification in the Berlin Questionnaire model.

After adjustment for age, sex, BMI, current smoking status, alcohol use, obesity, lung disease, diabetes mellitus and hypertension, BQ-defined high-risk OSA remained independently associated with moderate-to-severe CAC (adjusted OR 2.74, 95% CI 1.29–5.78, *p* = 0.008). Unadjusted and multivariable logistic regression results for the BQ model are summarized in Table 2, and the adjusted associations are illustrated in Figure 3.

**Figure 4:**
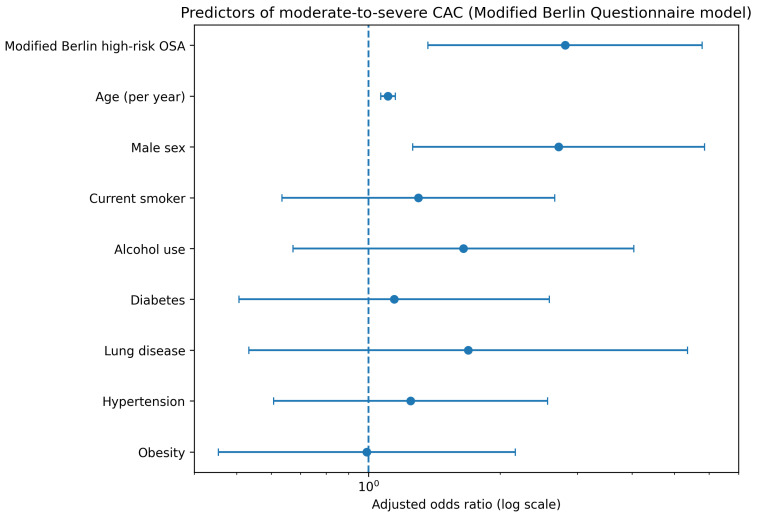
Predictors of moderate-to-severe coronary artery calcification in the modified Berlin Questionnaire model.

In univariate analyses, mBQ-defined high-risk OSA was also significantly associated with CAC > 100 (46.4% vs. 13.6%, *p* < 0.001). This association persisted after multivariable adjustment, with mBQ-defined high-risk OSA remaining an independent predictor of moderate-to-severe CAC (adjusted OR 2.62, 95% CI 1.27–5.41, *p* = 0.009). Detailed regression results for the mBQ model are presented in Table 2, with corresponding forest plots shown in Figure 4.

Across both models, older age and male sex were consistently associated with moderate-to-severe CAC, while the association between OSA risk and CAC remained robust regardless of the OSA risk definition used.

### 3.4. Association Between Snoring Intensity and Coronary Artery Calcification

**Figure 5:**
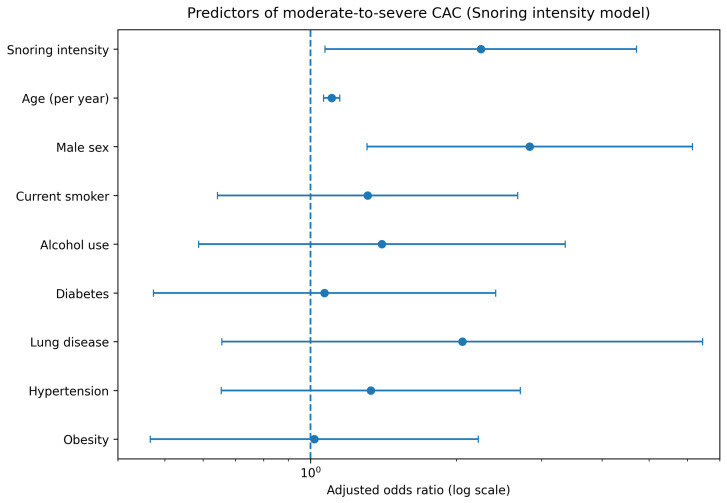
Association between snoring intensity and moderate-to-severe coronary artery calcification.

In symptom-focused analyses, snoring intensity was evaluated as an indicator of OSA-related symptom burden independent of composite OSA risk definitions. In univariate analysis, increased snoring intensity was significantly associated with the presence of moderate-to-severe CAC. After multivariable adjustment for age, sex, current smoking status, alcohol use, diabetes mellitus, lung disease, hypertension, and obesity, snoring intensity remained independently associated with CAC > 100 (adjusted OR 2.25, 95% CI 1.07–4.72, *p* = 0.032). Detailed regression results are presented in Table 3, and the adjusted association is illustrated in Figure 5.

## 4. Discussion

In this prospective cross-sectional study of adults without known cardiac disease undergoing routine non-contrast chest CT, we demonstrated that questionnaire-defined high-risk obstructive sleep apnea (OSA) is independently associated with moderate-to-severe coronary artery calcification (CAC), defined as an Agatston score > 100. This association remained statistically significant after adjustment for major cardiovascular risk factors, including age, sex, smoking, diabetes mellitus, hypertension, obesity, alcohol use, and chronic lung disease. Importantly, the relationship was consistent across both the standard Berlin Questionnaire (BQ) and a modified version excluding hypertension and obesity components. In addition, increased snoring intensity independently predicted moderate-to-severe CAC, suggesting that OSA-related symptom burden itself carries cardiovascular relevance. Collectively, these findings support a clinically meaningful link between OSA risk and subclinical coronary atherosclerosis detectable on routine thoracic imaging. Our findings are broadly consistent with previous imaging-based studies demonstrating an association between obstructive sleep apnea and coronary atherosclerosis. Several investigations using coronary CT angiography or calcium scoring have reported higher plaque burden and elevated CAC scores among patients with OSA. In line with these observations, Macek et al. [33] demonstrated that individuals with OSA had a higher likelihood of significant CAC assessed non-invasively using calcium scoring techniques. The present study extends this body of evidence by demonstrating that even questionnaire-based OSA risk—without formal polysomnographic confirmation—is independently associated with moderate-to-severe CAC detected incidentally on routine chest CT.

### 4.1. OSA as a Cardiovascular Risk Multiplier

OSA is increasingly recognized not merely as a sleep disorder but as a systemic condition characterized by chronic intermittent hypoxia, sleep fragmentation, and sustained sympathetic activation [3,6]. These pathophysiological perturbations initiate oxidative stress, systemic inflammation, endothelial dysfunction, and metabolic dysregulation—core processes in atherogenesis [4,5,7,8,9]. Endothelial dysfunction represents an early and potentially reversible stage of vascular injury, preceding structural plaque formation and calcification.

Rather than functioning as an isolated risk factor, OSA may act as a cardiovascular “risk amplifier.” Although patients classified as high risk for OSA were older on average, the association between OSA risk and moderate-to-severe CAC remained statistically significant after adjustment for age in multivariable models. Traditional models emphasize age, sex, smoking, diabetes, hypertension, and dyslipidemia; however, OSA introduces cyclical hypoxemia and autonomic instability that may accelerate the progression of vascular injury in susceptible individuals. The persistence of the association between OSA risk and moderate-to-severe CAC after multivariable adjustment suggests that OSA may lower the threshold at which vascular stress translates into calcified plaque burden. In this framework, OSA interacts synergistically with classical cardiometabolic risk factors, amplifying cumulative atherosclerotic exposure over time.

### 4.2. Intermittent Hypoxia and Coronary Calcification Biology

The repetitive hypoxia–reoxygenation cycles characteristic of OSA resemble ischemia–reperfusion injury at a microvascular level, triggering inflammatory signaling pathways and reactive oxygen species generation [4,7,8,9]. These processes promote vascular smooth muscle cell proliferation and may facilitate osteogenic differentiation within the arterial wall, contributing to calcification. Although calcification can represent plaque stabilization, total CAC burden reflects cumulative atherosclerotic exposure and strongly predicts future cardiovascular events [13,37].

Even after adjustment for obesity, hypertension, and diabetes mellitus, the association between OSA risk and CAC remained robust. This finding suggests that mechanisms intrinsic to sleep-disordered breathing—particularly nocturnal hypoxic burden and sleep fragmentation—may exert vascular effects beyond classical metabolic mediators. Over time, repeated hypoxic stress may contribute to both plaque development and its calcific transformation.

### 4.3. Silent Convergence of OSA and Subclinical Atherosclerosis

Both OSA and subclinical coronary atherosclerosis are frequently silent conditions. Many individuals with moderate-to-severe OSA remain undiagnosed for years, and patients with substantial CAC burden often lack overt cardiac symptoms. The convergence of these two silent pathologies represents a clinically important but underrecognized overlap.

From a practical standpoint, both CAC assessment and questionnaire-based OSA screening are relatively easy to implement within routine clinical workflows. The BQ can typically be completed by patients within a few minutes while waiting for imaging procedures, and automated calcium-scoring software enables rapid CAC quantification by radiologists. Integrating these simple tools into routine chest CT workflows may therefore represent a pragmatic strategy for identifying individuals with increased cardiovascular risk.

In our cohort, nearly one-quarter of patients exhibited moderate-to-severe CAC despite no known cardiac disease. Among high-risk OSA individuals, the prevalence was substantially higher. This suggests that undetected sleep-disordered breathing may coexist with—and potentially contribute to—subclinical coronary atherosclerosis long before clinical events manifest. Recognizing this “silent convergence” may improve early detection strategies and reduce delays in preventive intervention.

### 4.4. Opportunistic Imaging as a Platform for Integrated Risk Assessment

CAC is a well-established marker of future cardiovascular risk independent of traditional factors [8]. Increasing evidence supports the prognostic value of incidental CAC detected on non-gated chest CT [15,16,17,18,19,34]. Because chest CT is widely performed for non-cardiac indications, it offers a unique opportunity for opportunistic cardiovascular risk assessment without additional radiation exposure or cost.

Our findings extend this paradigm by demonstrating that questionnaire-based OSA risk identifies a subgroup with disproportionately higher CAC burden within the general chest CT population. Moderate-to-severe CAC (Agatston score > 100) is associated with significantly increased long-term cardiovascular risk [13,14]; therefore, the observed 2.6–2.7-fold increase in adjusted odds among high-risk OSA patients represents clinically meaningful risk enrichment.

From a translational perspective, integrating OSA risk assessment with incidental CAC detection may enhance precision prevention strategies. Patients with high OSA risk and moderate-to-severe CAC may warrant intensified risk factor management, closer cardiovascular surveillance, and formal sleep evaluation.

### 4.5. Snoring Intensity and Symptom-Based Risk Stratification

The independent association between snoring intensity and CAC underscores the potential cardiovascular relevance of symptom burden. Snoring reflects upper airway resistance and vibratory mechanical stress. Experimental data suggest that vibration may induce endothelial dysfunction and inflammatory responses in adjacent vascular structures [38], and acoustic snoring characteristics have been linked to surrogate vascular markers [39,40].

Although snoring alone does not establish an OSA diagnosis, its severity may reflect underlying nocturnal airflow limitation and intermittent hypoxia. Our findings indicate that symptom intensity—not only composite risk classification—may carry incremental cardiovascular information. Structured sleep history taking remains an accessible and low-cost clinical tool and may complement imaging findings in cardiovascular risk stratification.

### 4.6. Multidisciplinary Implications

These findings also highlight the importance of closer collaboration between radiology, sleep medicine, and preventive cardiology. Radiologists are often the first clinicians to detect incidental CAC on chest CT, yet sleep-related symptoms are rarely evaluated in radiological practice. Conversely, sleep clinics may not routinely review prior thoracic imaging findings when assessing patients with suspected OSA. Integrating sleep risk assessment with opportunistic imaging findings may therefore enhance multidisciplinary cardiovascular risk evaluation.

Our results support a collaborative model in which incidental CAC reporting prompts review of sleep-related symptoms, and high OSA risk prompts attention to available imaging data. Such multidisciplinary integration could enhance coordinated care and optimize early prevention strategies.

### 4.7. Precision Prevention and Systems-Level Considerations

Precision prevention aims to identify high-risk individuals before clinical events occur. Combining questionnaire-based OSA screening with opportunistic CAC detection represents a pragmatic and scalable approach aligned with value-based care principles. Both tools are inexpensive, widely available, and easily implemented in routine workflows.

High-risk OSA patients in our cohort constituted a subgroup enriched for subclinical coronary atherosclerosis. Identifying this subgroup may refine decisions regarding lipid-lowering therapy intensity, lifestyle counseling, and further cardiovascular evaluation. Future research integrating CAC burden, objective sleep metrics, and emerging biomarkers may further improve individualized risk prediction.

Importantly, OSA is a potentially modifiable condition. Although large randomized trials have shown mixed results regarding secondary cardiovascular prevention with CPAP therapy [41], improvements in endothelial function and autonomic regulation have been demonstrated [42]. Detecting OSA in individuals with subclinical atherosclerosis may represent an earlier and potentially more effective window for intervention.

Future longitudinal studies are needed to determine whether individuals with both elevated OSA risk and moderate-to-severe CAC represent a particularly high-risk subgroup for cardiovascular events. In addition, studies incorporating objective sleep measurements—such as polysomnography or home sleep apnea testing—may further clarify the mechanistic relationship between nocturnal hypoxic burden and the progression of coronary calcification.

### 4.8. Strengths and Limitations

This study has several strengths, including prospective design, evaluation of a real-world imaging cohort without known cardiac disease, standardized Agatston scoring, and comprehensive multivariable adjustment. The parallel assessment of standard and modified OSA risk definitions strengthens the robustness of the findings.

Limitations include the cross-sectional design, which precludes causal inference. OSA risk was assessed using questionnaire-based screening rather than polysomnography. Therefore, recall bias and the known sensitivity and specificity limitations of the BQ may have influenced the classification of OSA risk. CAC quantification was performed on non-gated chest CT, which may introduce measurement variability; however, clinically meaningful thresholds have demonstrated good agreement with gated protocols [15,16,17,18,19,37]. Residual confounding cannot be entirely excluded. Finally, questionnaire administration depended on staff availability during routine clinical workflow, which may have introduced selection bias. Patients with severe illness, cognitive impairment, or communication difficulties may have been less likely to complete the questionnaire.

## 5. Conclusions

In adults without known cardiac disease undergoing routine non-contrast chest CT, questionnaire-defined high-risk OSA is independently associated with moderate-to-severe coronary artery calcification. The consistency of this association across standard and modified risk definitions, together with the independent contribution of snoring intensity, supports a pathophysiologically relevant link rather than simple confounding by traditional cardiometabolic factors. These findings highlight the emerging role of opportunistic thoracic imaging in integrated cardiovascular risk assessment and reinforce the concept that sleep health is an integral component of cardiovascular health. Future longitudinal studies are warranted to determine whether targeted OSA management can influence the trajectory of coronary calcification and improve long-term cardiovascular outcomes.

## Figures and Tables

**Table 1:** Baseline characteristics according to the Berlin Questionnaire–defined OSA risk.

Variable	Low-Risk OSA (n = 166)	High-Risk OSA (n = 102)	*p* Value
Age (years)	55.9 ± 13.6	65.6 ± 11.1	<0.001
BMI (kg/m^2^)	25.9 ± 5.0	28.1 ± 5.4	0.001
Male sex, n (%)	58 (34.9%)	53 (52.0%)	0.009
Current smoker, n (%)	76 (45.8%)	50 (49.0%)	0.697
Alcohol use, n (%)	38 (22.9%)	20 (19.6%)	0.613
Diabetes mellitus, n (%)	22 (13.3%)	29 (28.4%)	0.004
Lung disease, n (%)	4 (2.4%)	15 (14.7%)	<0.001
Hypertension, n (%)	38 (22.9%)	60 (58.8%)	<0.001
Obesity, n (%)	24 (14.5%)	35 (34.3%)	<0.001
Moderate–severe CAC (Agatston > 100), n (%)	20 (12%)	44 (43.1%)	<0.001

Values are presented as mean ± standard deviation or number (percentage). *p* values were calculated using the independent samples *t* test for continuous variables and the χ^2^ test for categorical variables. Abbreviations: BMI, body mass index; CAC, coronary artery calcification; OSA, obstructive sleep apnea.

**Table 2:** Unadjusted and multivariable-adjusted logistic regression analyses evaluating the association between OSA and CAC (Agatston score > 100).

Variable	Unadjusted OR (95% CI)	*p* Value	Adjusted OR (95% CI)	*p* Value
Berlin Questionnaire high-risk OSA	5.53 (3.00–10.20)	<0.001	2.74 (1.29–5.78)	0.008
Modified Berlin Questionnaire high-risk OSA	5.52 (2.96–10.31)	<0.001	2.62 (1.27–5.41)	0.009
Age (per year)	–	–	1.11 (1.07–1.15)	<0.001
Male sex	–	–	3.06 (1.45–6.46)	0.003
Current smoker	–	–	1.45 (0.71–2.95)	0.39
Alcohol use	–	–	1.64 (0.67–4.02)	0.33
Diabetes mellitus	–	–	0.81 (0.34–1.92)	0.82
Lung disease	–	–	1.65 (0.52–5.22)	0.41
Hypertension	–	–	1.17 (0.56–2.44)	0.69
Obesity	–	–	0.96 (0.44–2.10)	0.92

Abbreviations: CAC, Coronary Artery Calcification; CI, Confidence Interval; OR, Odds Ratio; OSA, Obstructive Sleep Apnea.

**Table 3:** Unadjusted and multivariable-adjusted logistic regression analyses evaluating the association between snoring intensity and moderate-to-severe coronary artery calcification (Agatston score > 100).

Variable	Unadjusted OR (95% CI)	*p* Value	Adjusted OR (95% CI)	*p* Value
Snoring intensity	2.87 (1.45–5.68)	0.002	2.25 (1.07–4.72)	0.032
Age (per year)	–	–	1.11 (1.06–1.15)	<0.001
Male sex	–	–	2.84 (1.31–6.15)	0.008
Current smoker	–	–	1.31 (0.64–2.68)	0.456
Alcohol use	–	–	1.41 (0.59–3.36)	0.445
Diabetes mellitus	–	–	1.07 (0.47–2.41)	0.872
Lung disease	–	–	2.06 (0.66–6.45)	0.216
Hypertension	–	–	1.33 (0.65–2.71)	0.429
Obesity	–	–	1.02 (0.47–2.22)	0.965

Abbreviations: CI, Confidence Interval; OR, Odds Ratio.

## Data Availability

Data collected for the study, including de-identified individual participant data, will be made available within 6 months after the publication of this article for academic purposes (e.g., meta-analyses), upon request to the corresponding author (yuksel.peker@lungall.gu.se), and with a signed data access agreement.
